# Alterations in histamine responses between juvenile and adult urinary bladder urothelium, lamina propria and detrusor tissues

**DOI:** 10.1038/s41598-020-60967-7

**Published:** 2020-03-05

**Authors:** Zane Stromberga, Russ Chess-Williams, Christian Moro

**Affiliations:** Centre for Urology Research, Faculty of Health Sciences and Medicine, Bond University, Queensland, 4229 Australia

**Keywords:** Bladder, Ageing

## Abstract

Inflammatory mediators may have a role in various lower urinary tract disorders. Histamine is known to induce significant increases in both the tension and frequency of spontaneous phasic contractions in both urothelium with lamina propria (U&LP) and detrusor muscle via the activation of H1 receptor in juvenile animal models. However, it is unclear whether age affects these contractile responses to histamine. This study assessed the histamine receptor subtypes mediating contraction in juvenile and adult porcine bladders and compared the urothelium with lamina propria and detrusor responses to histamine. Isolated tissue bath studies were conducted using strips of porcine U&LP and detrusor obtained from juvenile (6 months) and adult (3 years) animals exposed to histamine receptor agonists and antagonists. Treatment with histamine (100 µM) in U&LP of juvenile animals caused increases in baseline tension by 47.84 ± 6.52 mN/g (p < 0.001, n = 51) and by 50.76 ± 4.10 mN/g (p < 0.001, n = 55) in adult animals. Furthermore, the frequency of spontaneous phasic contractions was significantly enhanced in response to histamine in U&LP of both juvenile and adult tissues (p < 0.001 for both age groups). Treatment with an H2 agonist in U&LP of juvenile animals decreased baseline tension by 13.97 ± 3.45 mN/g (n = 12, p < 0.05), but had no effect in adult animals. Inhibition of H1 receptors resulted in significantly reduced contractile responses of U&LP and detrusor to histamine in both juvenile and adult animals (p < 0.05). Treatment with an H2 receptor antagonist significantly enhanced contractions in juvenile preparations (n = 10, p < 0.05) but had no effect in adult preparations (n = 8). In detrusor, treatment with histamine (100 µM) in juvenile tissues showed a significantly higher increase in baseline tension of 19.10 ± 4.92 mN/g (n = 51) when compared to adult tissues exhibiting increases of 8.21 ± 0.89 mN/g (n = 56, p < 0.05). The increases in the baseline tension were significantly inhibited by the presence of H1 receptor antagonists in both juvenile and adult detrusor preparations. Treatment with either the H2 receptor antagonist or agonist in detrusor had no effect on both juvenile and adult tissues. Therefore, the histamine receptor system may play an essential role in the maintenance of bladder function or in bladder dysfunction observed in some lower urinary tract disorders.

## Introduction

Normal bladder function deteriorates throughout adult life, age appearing to have a direct effect on bladder sensation, contractility and the ability to postpone voiding^1^. Ageing is associated with several changes that occur in the urinary bladder, such as a reduction in bladder capacity^2^ and an increase in bladder sensitisation^3^. It is also linked with marked increases in the prevalence of lower urinary tract symptoms, such as urinary frequency, voiding difficulties, decreased bladder contractions (as seen in underactive bladder) or uninhibited bladder contractions (as seen in overactive bladder)^1^. Urinary frequency and incontinence are common in the general population, with a significant increase over the age of 65 years^4^. With an increasing ageing population worldwide^5^, the incidence of lower urinary tract symptoms is likely to rise. The mechanisms underlying age-related bladder dysfunction are largely unclear, however, it is known that the prevalence of these symptoms generally increases with age^6^. At this current moment, there are a limited number of studies exploring the physiology of the lower urinary tract throughout ageing.

Age-related changes in the bladder structure, voiding patterns and neurotransmitter release have been studied to some extent, but they often provide conflicting evidence. Specifically, contractile responses to endogenous chemicals, noradrenaline, ATP and 5-HT, were increased with age^7^. Relaxatory responses to an adrenoceptor agonist, isoproterenol, were significantly inhibited in comparison to younger age groups, although the authors found no significant age-related differences in the contractile responses to acetylcholine, prostaglandin F_2α_, angiotensin II, vasoactive intestinal polypeptide or KCl. Additional functional studies involving human tissue also revealed that there is a non-neuronal release of acetylcholine from both the human U&LP and detrusor, which increases during ageing^8^ capable of impacting the overall contractility of the bladder. Furthermore, in a mouse model Daly, *et al*.^9^ found that ageing was associated with increases in voiding frequency, the release of ATP, frequency of spontaneous detrusor contractions, contractile responses of detrusor to muscarinic and purinergic agonists and afferent nerve activity while urothelial acetylcholine release was reduced. However, the mechanisms underlying these observed functional changes with ageing are unknown, with some variation exhibited between *ex-vivo*^9^ and *in-vivo* animal and human models^10^. Based on these contractile changes in response to different endogenous chemicals and pharmaceutical agents, it is likely that other receptor systems that are capable of inducing contractile changes in the bladder may also be affected by age and therefore an important avenue to explore further.

One such system is the histaminic receptor system that is involved in the modulation of the urinary bladder contractions and spontaneous activity;^11^ however it remains unclear how ageing impacts this receptor system. Histamine exerts its function by binding to four different G protein-coupled receptors: H1, H2, H3 and H4^12^. Both H1 and H2 receptors are co-expressed in most tissues, including smooth muscle, epithelial tissue, neurons and on various white blood cells^13^. When histamine binds to H1 receptor, it couples G_q/11_ which stimulates phospholipase C (PLC) thereby initiating the generation of second messengers: inositol 1,4,5-triphosphate (IP3) and diacylglycerol (DAG)^14^. IP3 then leads to the release of calcium from the sarcoplasmic reticulum within the cell thereby initiating the process of smooth muscle contraction. The maintenance of these contractions is dependent on both internal and external sources of calcium. Activation of the H2 receptor couples G_s_ protein triggering adenylyl cyclase (AC) activation and accumulation of cAMP^15^ leading to calcium decrease in the cell^16^ and relaxation of the smooth muscle. Both H3 and H4 receptors couple to G_i/o_ that inhibit AC thereby decreasing the cAMP levels. H4 receptors are also involved in mediating calcium mobilisation in mast cells and the release of calcium from the intracellular calcium stores^17^ which has been suggested as the mechanism responsible for mast cell accumulation in inflamed tissue.

Immunohistochemical analysis of cultured human detrusor cells identified the presence of all four histamine receptor subtypes: H1, H2, H3 and H4^18^. However, no immunohistochemical studies have been performed to determine the presence of these receptor subtypes in urothelium or lamina propria of the urinary bladder. Administration of histamine has previously shown a contractile response via the activation of the H1 receptors in isolated guinea pig^19–21^ and rabbit^22^ bladders. Furthermore, our previous findings^11^ established functional responses to histamine not only in detrusor but also in urothelium with lamina propria layer of the urinary bladder via stimulation of H1 and H2 receptors.

There are currently no studies that have investigated the impacts of ageing on the contractile responses to histamine in the urinary bladder. In the mouse brain, age is associated with significant changes in the histamine receptor mRNA levels and subsequent reduction in the expression of the H1, H2 and H3 receptors^23^. In the human brain, similar observations were noted, where the H1 receptor subtype showed significant decreases with age^24^. The ability of histamine to induce vasodilation via the activation of H1 and H2 receptors is also altered with age^25^. The authors demonstrated that ageing has a direct effect on the signal transduction pathway activated via the H2 receptor while maintaining the function of H1 receptor. Furthermore, functional studies have shown  that decreases in the histamine-stimulated AC activity occur in aged rabbits, appearing to be represented as reductions to maximal responses rather than an alteration in receptor affinity^26^ and K^+^-induced histamine release is lowered in *in vitro* experiments involving aged rat hypothalamus^27^.

As a potent inflammatory mediator released from mast cells, histamine may play a key role in the pathogenesis of various lower urinary tract disorders. Several research studies have reported signs of inflammation in urinary bladder biopsies obtained from patients suffering from bladder overactivity^28,29^ and established a central role of inflammation in the pathogenesis of IC/BPS^30^. An increased expression of monocyte chemoattractant protein-1 (MCP-1), which stimulates the release of inflammatory mediators from mast cells^31,32^, has been suggested as a contributor to the inflammation observed in the tissue^33^. Specifically, in the urinary bladder, mast cells can be found in all layers of the bladder wall, including urothelium, lamina propria and detrusor smooth muscle^34,35^. When urothelial cells of the bladder are damaged or stressed, they are capable of releasing ATP^36^, IL-33^37^ and β -defensins that directly trigger the degranulation of mast cells and subsequent release of pro-inflammatory mediators, such as histamine, prostaglandins, proteases and cytokines^38^.

Increased cellular responsiveness to histamine is a key feature of many inflammatory conditions^39^. However, it is unknown whether the histamine receptor system directly mediates contractions of the bladder dome and is associated with bladder pathologies. Histamine is known to induce significant increases in both the tension and frequency of spontaneous phasic contractions in both the urothelium with lamina propria and in the underlying detrusor muscle via the activation of the H1 receptors^11^. It also enhances the mechanosensitivity of nearby afferent nerves to bladder distension, resulting in an increased neuronal activation in the spinal cord^40^. In addition, this effect may work in conjunction with other inflammatory-cell released chemicals such as 5-HT^41^ to induce or maintain various lower urinary tract disorders. However, it is unclear how age affects the contractile responses to histamine.

Based on prior histamine receptor expression research, it is hypothesised that responses from stimulating the H1 receptor will be reduced with ageing in both U&LP and detrusor smooth muscle. It is also hypothesised that stimulation of the H2 receptor, shown to be involved in inhibitory responses in urothelium with lamina propria^11^, will have a more significant impact on juvenile tissues when compared to adult tissues. The aim of this study was to compare responses to histamine in both U&LP and detrusor tissues from porcine juvenile and adult bladders.

## Materials and Methods

### Tissue source and acquisition

Urinary bladders of crossbred Large-White-Landrace-Duroc (*Suf scrofa domestica*) pigs were used as the tissue in this study. Juvenile samples were obtained from prepubescent pigs aged 6 months old at 80 kg live-weight. Adult tissues were taken from sow animals, aged 2–3 years old at ~200 kg live-weight. All bladders were obtained from the local abattoir after slaughter for the routine commercial provision of food with no animals bred, harmed, culled, interfered, or interacted with as part of this research project. As such, animal ethics approval was not required (Queensland Government, 2016).

Immediately after the slaughter of pigs at the abattoir, the bladders were removed and directly placed in a cold Krebs-bicarbonate solution at 4 °C (NaCl 118.4 mM, NaHCO_3_ 24.9 mM, CaCl_2_ 1.9 mM, MgSO_4_ 2.41 mM, KCl 4.6 mM, KH_2_PO_4_ 1.18 mM and D-glucose 11.7 mM). After collection, the container with porcine bladders was stored in a portable cooler and transported back to Bond University’s research laboratory to be set up within 3 hours of the animal’s slaughter. Upon return, tissues were prepared for experiments by removing the ureters and urethra, serosa and arteries on the outside of the bladder.

### Tissue preparation

Intact strips of the urinary bladder were horizontally removed from the anterior base wall of the bladder dome region. Urothelium with lamina propria was dissected from the underlying detrusor layer, consistent with methods carried out in past studies^11,42^. Throughout the preparation and dissection stage, tissue strips were constantly washed with a cold Krebs-bicarbonate solution.

Once the tissues were dissected, adjacent strips of U&LP and detrusor (each strip is 10 mm in length and 5 mm in width) were mounted vertically between an isometric force transducer (MCT050/D, ADInstruments, Castle Hill, Australia) and a fixed hook in a 10 mL organ bath (Labglass, Brisbane, Australia), and superfused with Krebs-bicarbonate solution (NaCl 118.4 mM, NaHCO_3_ 24.9 mM, CaCl_2_ 1.9 mM, MgSO_4_ 2.41 mM, KCl 4.6 mM, KH_2_PO_4_ 1.18 mM and D-glucose 11.7 mM) at 37 °C and carbogen (95% oxygen and 5% carbon dioxide).

After mounting of tissues, strips of U&LP and detrusor were washed three times with warm Krebs-bicarbonate solution and the tension manually adjusted to 2 g on a moveable transducer positioner with a fine adjustment lever. As such, baseline tension for each experiment was calculated from this 2 g set-point. Tissues were then left to equilibrate for 30 min in the absence (control) and presence (experimental) of a specific histamine receptor antagonist. After the equilibration period, a single dose of histamine was added to both control and experimental tissues. The increases in baseline tension and in the frequency and amplitude of spontaneous phasic contractions were recorded simultaneously through an isometric force transducer on a Powerlab system using LabChart v7 software (MCT050/D, ADInstruments, Castle Hill, Australia). The viability of the tissue was ensured by adding a single dose of carbamoylcholine chloride (10 µM, Sigma Aldrich, Missouri, USA) to all tissues at the very end of each experiment. U&LP or detrusor strips that did not respond to carbamoylcholine chloride were deemed as non-viable and were not included in the analysis. At the conclusion of each experiment, tissues were removed from each organ bath and measured on a weighing scale to an accuracy of 1 mg (0.001 g).

### Measurements and data collection

Measurements of the baseline tension and the frequency and amplitude of spontaneous phasic contractions were taken before the agonist was added and during peak contractile response after the addition of the agonist. The frequency of spontaneous contractions was measured from the total number of phasic waves occurring over 2–3 minutes, calculated as an average, and expressed as contractions per minute (cpm). The amplitude of each contraction was measured as the tension differences between the averaged lowest and highest point of each phasic wave. Baseline tension was measured from the lowest point of each spontaneous phasic contraction before treatment with an agonist and during peak contractile response. Changes in both baseline tensions and the amplitudes of spontaneous phasic contractions were expressed as Newton force per gram tissue weight (mN/g).

### Pharmaceutical agents

Histamine dihydrochloride, amthamine dihydrobromide, pyrilamine maleate salt, cimetidine, thioperamide maleate salt, indomethacin, atropine, and Nω-Nitro-L-arginine were obtained from Sigma-Aldrich (Missouri, USA), and fexofenadine hydrochloride, αβ-methylene ATP (sodium salt) and cyproheptadine hydrochloride hydrate from Cayman Chemicals (Michigan, USA). Concentrations chosen for the agonists and antagonists were selected based on their selectivity at each receptor and consistent with concentrations used in previous studies^11^.

### Statistical analysis

Data were graphed and analysed using GraphPad Prism version 8.3 for Windows (GraphPad Software, La Jolla California USA) and results expressed as the mean change ± SEM. Within experiments using only juvenile, or only adult tissues, data were calculated as the total change between the control and its paired experimental sample. For comparisons between age groups, data comparing juvenile with adult samples were calculated from the averaged changes between each tissue’s responses when placed under identical experimental parameters. All responses were compared using a Student’s two-tailed *t*-test, with p < 0.05 considered as statistically significant. A *paired* Student’s two-tailed *t*-test was applied to tissues with direct controls (within each juvenile or adult experiment) and an *unpaired* Student’s two-tailed *t*-test was applied to make comparisons between juvenile and adult groups.

## Results

### Histamine agonists for stimulating spontaneous U&LP activity in juvenile and adult tissues

In the absence of any stimulation, strips of U&LP naturally developed spontaneous phasic contractions. In juvenile animals, spontaneous contractions occurred at 3.37 ± 0.05 cycles per minute (cpm, n = 159) with an amplitude of 32.63 ± 1.81 mN/g (n = 159). In adult animals, these contractions occurred at 3.37 ± 0.11 cpm (n = 59) with an amplitude of 34.88 ± 3.79 mN/g (n = 59). There was no significant difference in the frequency or amplitude of spontaneous phasic contractions between juvenile (n = 159) and adult (n = 59) tissues.

When histamine (100 µM) was added to U&LP strips of juvenile animals, the frequency of spontaneous phasic contractions increased by 1.29 ± 0.26 cpm (p < 0.001, n = 51) and the amplitude of each contraction decreased by 5.54 ± 1.60 mN/g (p < 0.001, n = 51). In U&LP strips of adult animals, an increase to the frequency of spontaneous phasic contractions occurred at 1.18 ± 0.16 cpm (p < 0.001, n = 55) and decrease in the amplitude of these contractions by 10.05 ± 2.06 mN/g (p < 0.001, n = 55). The increases in the baseline tension and in the frequency and amplitude of spontaneous phasic contractions in response to histamine (100 µM) were not significantly different between juvenile (n = 51) and adult (n = 55) tissues.

Treatment with H2 receptor agonist amthamine had no effect on the frequency or on the amplitude of the spontaneous phasic contractions in either juvenile or adult U&LP preparations.

### Histamine agonists for stimulating spontaneous detrusor activity in juvenile and adult tissues

In detrusor preparations of juvenile animals, spontaneous activity in the absence of any stimulation was present in 13% of preparations with an average frequency of 2.06 ± 0.30 cpm and amplitude of 18.29 ± 3.07 mN/g (n = 7). After the addition of histamine (100 µM), the frequency was significantly enhanced, increasing to 4.47 ± 0.69 cpm (n = 7, p < 0.01) with an amplitude of 29.07 ± 6.40 mN/g (n = 7, NSD). In adult preparations, this activity was present in 30% of all preparations (n = 17) with an average frequency of 1.95 ± 0.15 cpm and amplitude of 14.26 ± 2.15 mN/g. In response to histamine (100 µM), frequency of spontaneous contractions was significantly enhanced to 2.39 ± 0.16 cpm (n = 17, p < 0.01) with an amplitude of 24.22 ± 4.36 mN/g (n =17, p < 0.05). There were no significant differences in the frequency or amplitude of spontaneous phasic contractions exhibited by detrusor preparations of both juvenile and adult animals before the treatment with histamine. During peak response to histamine (100 µM), strips of detrusor from juvenile animals (n = 7) exhibited a significantly higher frequency of spontaneous contractions when compared to adult tissues (n = 17, p < 0.001). The amplitude of spontaneous contractions exhibited during peak response to histamine (100 µM) was not significantly different between the two age groups. Spontaneous phasic activity was not present at any point during the experiment in 35% of juvenile preparations and in 34% (n = 18) of adult preparations (n = 19).

In those detrusor strips that did not develop spontaneous activity before the addition of the agonist, treatment with histamine (100 µM) initiated contractions in 59% (n = 26) of preparations of juvenile animals with a frequency of 2.90 ± 0.20 cpm and amplitude of 24.46 ± 8.40 mN/g. In adult animals, contractions were initiated in 51% of preparations (n = 20) with an average frequency of 2.83 ± 0.32 cpm and amplitude of 14.79 ± 1.61 mN/g. There were no significant differences in the frequency and amplitude of spontaneous contraction developed after treatment with histamine (100 µM) between the two age groups.

### Histamine agonists for stimulating contractions in U&LP and detrusor in juvenile and adult tissues

**Figure 1:**
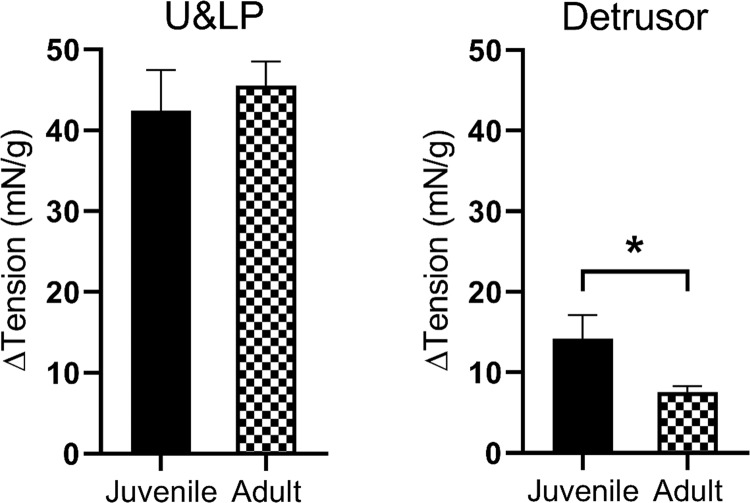
Influence of age on the increases in baseline tension in response to histamine (100 µM) in U&LP (*left*) and detrusor (*right*) preparations. *p < 0.05.

When histamine (100 µM) was added to U&LP strips of juvenile animals, baseline tension increased by 47.84 ± 6.52 mN/g (p < 0.001, n = 51, Fig. 1). Similarly, the addition of histamine (100 µM) to U&LP strips of adult animals increased the baseline tension by 50.76 ± 4.10 mN/g (p < 0.001, n = 55, Fig. 1). In detrusor preparations, juvenile tissues showed significantly greater (p < 0.05) increases in baseline tension of 19.10 ± 4.92 mN/g (n = 51) when compared to tissues from adult animals which exhibited increases of 8.21 ± 0.89 mN/g (n = 56, Fig. 1) in response to histamine (100 µM).

**Figure 2:**
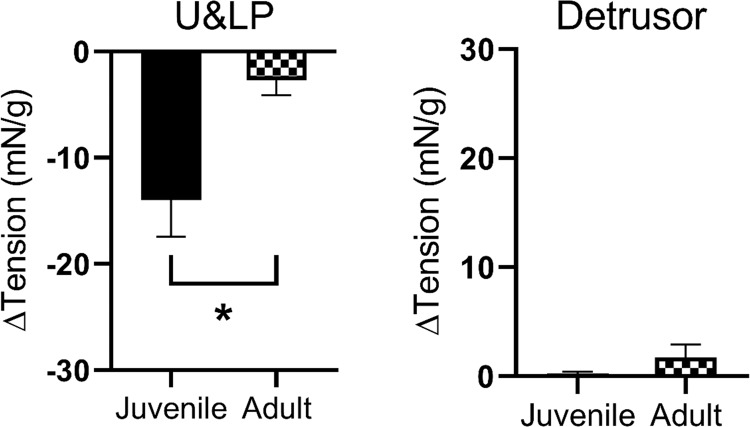
Influence of age in response to H2 agonist amthamine (1 µM) in U&LP (*left*) and detrusor (*right*) preparations. *p < 0.05.

In bladders of juvenile animals, U&LP responses to the H2 agonist amthamine (1 µM) resulted in a baseline tension decrease of 13.97 ± 3.45 mN/g (n = 12, p < 0.05). The preparation of juvenile U&LP tissues showed a significantly greater reduction in the baseline tension in response to amthamine (1 µM) when compared to adult U&LP tissues (p < 0.05, Fig. 2). In detrusor preparations, the addition of amthamine (100 µM) in both juvenile (n = 4) and adult (n = 8) tissues had no effect on the baseline tension (Fig. 2).

### Receptor-selective antagonists in U&LP preparations of juvenile and adult tissues

The influence of three different H1 antagonists and their baseline tension responses to histamine was compared between juvenile and adult tissues. Strips of U&LP treated with the H1 receptor antagonists pyrilamine (30 nM), fexofenadine (1 μM) and cyproheptadine (30 nM) showed significant inhibition of the increases in the baseline tension in responses to histamine in both juvenile and adult preparations (100 μM, p < 0.01 for all, Table 1). None of the baseline tension responses to histamine (100 μM) in the presence of any of the three H1 antagonists were significantly different between juvenile or adult tissues (unpaired Student’s *t*-test, p = NSD for all).

Treatment with cimetidine (1 μM, H2 antagonist) in juvenile U&LP tissues significantly enhanced increases in the baseline tension by 19.05 ± 8.32 mN/g (n = 10, p < 0.05) in response to histamine (100 μM) but had no effect in adult tissues (n = 8). Treatment with thioperamide (1 μM, H3/H4 antagonist) in juvenile tissues caused significantly enhanced increases in the baseline tension of 27.33 ± 8.67 mN/g (n = 6, p < 0.05) in response to histamine (100 μM). The opposite effect was observed in adult tissues in response to histamine (100 μM), where increases in the baseline tension were significantly inhibited by 17.64 ± 7.22 mN/g (n = 8, p < 0.05, Table 1). However, upon further investigation involving a combination of histamine receptor antagonists, it was revealed that thioperamide had no effect baseline tension in response to histamine (100 μM) in both juvenile and adult tissues.

**Table 1:** Comparison of U&LP changes to baseline tension (mN/g) in response to histamine (100 µM) in the absence and presence of histamine receptor antagonists (mean change ± SEM) in juvenile and adult urinary bladders.

Antagonist	Juvenile (mN/g)			Adult (mN/g)		
	Absence	Presence	n	Absence	Presence	n
Pyrilamine	34.82 ± 11.09	6.39 ± 5.74***	14	34.52 ± 4.14	17.56 ± 2.81**	14
Fexofenadine	44.63 ± 11.78	−1.63 ± 1.01**	8	46.03 ± 4.44	−0.59 ± 2.21**	8
Cyproheptadine	59.56 ± 18.97	21.00 ± 8.11*	8	66.18 ± 7.97	28.46 ± 6.04**	8
Cimetidine	61.70 ± 22.26	80.75 ± 28.12*	10	41.40 ± 4.45	50.81 ± 7.85	8
Thioperamide	59.92 ± 14.35	87.25 ± 14.71*	6	51.47 ± 9.78	33.82 ± 5.19*	8

*p < 0.05, **p < 0.01, ***p < 0.001. Paired Student’s *t*-test.

In both juvenile and adult preparations, increases in the frequency of spontaneous phasic contractions were significantly inhibited in response to histamine (100 μM) when the tissues were treated with pyrilamine (30 nM, p < 0.05 for both). Treatment with fexofenadine (1 µM) caused complete inhibition to the increases to the baseline tension in response to histamine (100 µM) in both juvenile (n = 8, p < 0.01) and adult tissues (n = 8, p < 0.01). In tissues from juvenile animals, cyproheptadine (30 nM) significantly inhibited increases in spontaneous activity (n = 8, p < 0.05) in response to histamine (100 µM), although this was not replicated in adult tissues (n = 8, Table 2).

Treatment with cimetidine (1 µM, H2 antagonist) had no effect on the frequency and amplitude of spontaneous phasic contractions in both juvenile tissues in response to histamine (100 μM). The addition of thioperamide (1 µM, H3/H4 antagonist) caused a significant inhibition in the frequency of spontaneous activity in tissues from adult animals (n = 8, p < 0.01), but had no effect in tissues obtained from juvenile animals (n = 6). None of the five histamine receptor antagonists had any influence on the amplitude of spontaneous phasic contractions in response to histamine (100 μM) in both juvenile and adult preparations (Table 2).

**Table 2:** Comparison of U&LP changes in the frequency of spontaneous phasic contractions (cpm) to histamine (100 µM) in the absence and presence of histamine receptor antagonists (mean change ± SEM) in juvenile and adult urinary bladders.

Antagonist	Juvenile (cycles per minute)			Adult (cycles per minute)		
	Absence	Presence	n	Absence	Presence	n
Pyrilamine	1.27 ± 0.29	0.23 ± 0.22**	12	0.89 ± 0.24	0.34 ± 0.10*	14
Fexofenadine	1.11 ± 0.28	−0.09 ± 0.14**	8	0.81 ± 0.15	−0.13 ± 0.09**	8
Cyproheptadine	1.87 ± 0.78	0.56 ± 0.28*	8	1.01 ± 0.29	0.77 ± 0.28	8
Cimetidine	1.77 ± 0.57	1.32 ± 0.75	10	1.40 ± 0.77	1.18 ± 0.26	8
Thioperamide	1.49 ± 0.63	1.76 ± 0.55	6	1.84 ± 0.41	0.63 ± 0.27**	8

*p < 0.05, **p < 0.01, ***p < 0.001. Paired Student’s two-tailed *t*-test.

The potential contributions of other receptor systems present in the U&LP to histamine-induced (100 μM) contractile responses were investigated. Neither baseline tension, the frequency or amplitude of spontaneous phasic contractions in response to histamine in both juvenile and adult strips of U&LP were affected by the presence of the muscarinic receptor antagonist atropine (1 µM), cyclooxygenase (COX) inhibitor indomethacin (5 µM), nitric oxide synthase inhibitor N_ω_-Nitro-L-arginine (L-NNA, 100 µM) or the P2X receptor desensitising agonist αβ-methylene ATP (αβm-ATP, 10 µM).

### Receptor-selective antagonists in detrusor preparations of juvenile and adult tissues

The presence of pyrilamine in juvenile detrusor preparations showed significantly greater inhibition of the contractile response to histamine (100 μM) when compared to adult tissues (p < 0.05). In juvenile preparations, increases in baseline tension were inhibited by 12.46 ± 3.88 mN/g (n = 14, p < 0.05) whereas in adult these increases were inhibited by 3.91 ± 1.73 mN/g (n = 14, p < 0.05, Table 3). Treatment with an alternative H1 antagonist fexofenadine inhibited increases in baseline tension to histamine (100 μM) by 9.61 ± 3.95 mN/g (n = 8, p < 0.05) in juvenile preparations and by 4.17 ± 1.33 mN/g (n = 8, p < 0.05) in adult. Similarly, the addition of H1 antagonist cyproheptadine caused inhibition of the contractile responses to histamine (100 μM) by 5.57 ± 1.22 mN/g (n = 8, p < 0.01) in juvenile tissues and by 3.00 ± 1.22 mN/g (n = 8, p < 0.05, Table 3) in adult tissue preparations. None of the baseline tension responses to histamine (100 μM) in the presence of any of the three H1 antagonists were significantly different between juvenile or adult detrusor tissues (unpaired Student’s two-tailed *t*-test, p = NSD for all).

Treatment with the H2 antagonist cimetidine in detrusor from juvenile animals (n = 8) demonstrated some inhibition of the contractile responses to histamine (100 μM), however, this response was not significant. In adult tissues (n = 8), cimetidine did not exert any effect on the increases in baseline tension in response to histamine (100 μM). Similarly, the H3 and H4 receptor dual antagonist thioperamide had no effect on the contractile responses to histamine (100 μM) in both juvenile (n = 6) and adult (n = 8) detrusor preparations (Table 3).

**Table 3:** Comparison of detrusor changes to baseline tension (mN/g) in response to histamine (100 µM) in the absence and presence of histamine receptor antagonists (mean change ± SEM) in juvenile and adult urinary bladders.

Antagonist	Juvenile (mN/g)			Adult (mN/g)		
	Absence	Presence	n	Absence	Presence	n
Pyrilamine	17.11 ± 4.93	4.65 ± 1.90*	8	11.20 ± 2.00	5.72 ± 1.15**	13
Fexofenadine	10.88 ± 3.65	1.27 ± 0.50*	8	4.53 ± 1.39	0.06 ± 0.13*	8
Cyproheptadine	7.06 ± 0.86	1.49 ± 0.66**	8	4.81 ± 1.38	1.52 ± 0.42*	8
Cimetidine	12.12 ± 3.28	5.48 ± 1.55	10	6.62 ± 2.02	8.30 ± 2.73	8
Thioperamide	18.88 ± 6.09	13.25 ± 3.33*	4	8.04 ± 2.02	5.64 ± 1.24	8

*p < 0.05, **p < 0.01, ***p < 0.001. Paired Student’s two-tailed *t*-test.

Baseline tension in response to histamine (100 μM) in both juvenile and adult strips of detrusor were not affected by the presence of the muscarinic receptor antagonist atropine (1 µM), cyclooxygenase (COX) inhibitor indomethacin (5 µM), nitric oxide synthase inhibitor N_ω_-Nitro-L-arginine (L-NNA, 100 µM) or P2X receptor desensitising agonist αβ-methylene ATP (αβm-ATP, 10 µM).

## Discussion

This study shows that ageing has varying effects on the contractile responses of U&LP or detrusor smooth muscle of the porcine bladder. This research has provided several novel findings that further our understanding of histamine’s influence on the bladder contractility throughout ageing. There are five main findings of this study. (1) Age does not affect increases in the baseline tension or the frequency of spontaneous phasic contractions in response to histamine in U&LP. (2) Maximal contractile responses in detrusor smooth muscle of adult tissues were significantly smaller when compared to contractions observed in juvenile animals. (3) Inhibition of the H2 receptors significantly enhanced increases to baseline tension in response to histamine in juvenile animals but had no effect in adult animals. (4) Stimulation of the H2 receptors causes relaxation of U&LP in juvenile but not in adult tissues. (5) Neither the muscarinic nor purinergic receptor systems are involved in the contractile response to histamine.

The current focus of pharmacological interventions for lower urinary tract symptoms has been primarily focused on muscarinic receptor antagonists due to the involvement of this receptor system in bladder voiding^43^. However, the use of antimuscarinics is associated with several side effects due to muscarinic receptor localisation throughout the body that limits their overall tolerability^44^. Due to the troublesome side effects, the long-term adherence and persistence to this treatment are generally low^45–47^ and declines rapidly after the initiation of the treatment^48^. Furthermore, the symptoms associated with uninhibited contractions that occur in patients with overactive bladder are considered to occur, in part, through stimulation of receptor pathways other than the muscarinic system^6^. The influence of different receptor systems within the U&LP has been of particular interest, specifically their impact on the spontaneous contractile activity that is known to occur during the filling phase^49,50^. These contractions may also play a role in the regulation of tone and the overall continence mechanisms, as previously hypothesised in the literature^50^.

Some of the chemicals that are capable of mediating contractions in the bladder include ATP^51^, 5-HT or serotonin^41^, prostaglandins^52^ and histamine^11^. Specifically, histamine exerts its function in both U&LP and detrusor smooth muscle via the activation of the H1 receptor as confirmed in guinea pig^19–21^, rabbit^22^ and pig^11^ animal models. The stimulation of the H2 receptor in porcine U&LP has recently been associated with inhibited contractions to histamine;^11^ however this finding was not present in the detrusor tissue. Having established a clear involvement of the histamine receptor system in mediating bladder contractions and spontaneous activity in tissues obtained from juvenile animals^11^, we undertook this study to determine how ageing impacts the responses to histamine and the involvement of the different histamine receptor subtypes. Our present study also confirmed the lack of involvement of purinergic or cholinergic receptor systems in the mediation of histamine-induced contractile responses that was first noted in Stromberga, *et al*.^11^ study.

This study found that maximal responses to histamine in detrusor were significantly lower in adult preparations when compared to juvenile preparations. Interestingly, a general decline in the contractile responses evoked by high potassium and muscarinic receptor agonist in adult rats has previously been reported in detrusor smooth muscle^53,54^. The mechanisms underlying this are unclear, but the urinary bladder contains elastic and collagen fibres that are responsible for the distensibility of the bladder wall and thus play a role in the generation of contractions and intravesical pressure^55^. As there is greater deposition of collagen observed in ageing bladder^56,57^, it is possible that the decrease in peak contractions occurred due to reduced compliance in adult detrusor.

Maximal contractile responses in U&LP and the frequency of spontaneous phasic contractions remained the same in both juvenile and adult tissues, indicating that the contractile properties of this layer were not affected by age. However, it appears that only the H1 receptor is functional in adult preparations of U&LP. In juvenile tissues, the H2 receptor has been shown to inhibit contractile responses to histamine^11^, yet neither stimulation nor inhibition of this receptor had any effect in adult tissues. This lack of response might have occurred due to a general reduction in the responsiveness of the H2 receptors within the U&LP layer, although this is yet to be confirmed. Based on our previous investigation on the histamine receptor system^11^, H3 and H4 receptor antagonist thioperamide exerts its function on other histamine receptor subtypes. As both H3 and H4 receptors have no direct contractile involvement in smooth muscle contractions^15^, it appears that this receptor agonist has a secondary effect on stimulating the H2 receptor in juvenile samples, thereby producing enhanced contractile responses. The involvement of this antagonist in enhancing contractions was ruled out using combination antagonist studies, where both H1 and H2 receptors were inhibited. It was determined that that thioperamide had no influence contractility of the U&LP or detrusor^11^. In adult samples, no responses were observed to the stimulation or inhibition of the H2 receptor. Thereby, it is likely that the inhibition of contractile responses in the presence of thioperamide observed in adult samples was a result of stimulating the H1 receptors.

Overall, the identification of a functional response to histamine agonists presents an interesting direction for future studies in the histaminic receptor signalling. The identification of which second-messenger systems are coupled to this response could provide more insight into the mechanisms underlying the contraction. In addition, future studies utilising immunocytochemical, immunohistochemical or radioligand binding assessments to determine the location, density, and prevalence of the histamine receptors would provide additional insights into this response.

## Conclusions

The histamine receptor system may play an important role in the maintenance of bladder function or in the stimulation of some contractile disease states in both juvenile and adult tissues. This study presents the possibility that a contributing factor to increased prevalence of bladder contractile disorders in an ageing population may be due to a general reduction in the responsiveness of histamine receptors or the lack of response to H2 stimulation.
